# Effect of thermal treatment on ZnO:Tb^3+^ nano-crystalline thin films and application for spectral conversion in inverted organic solar cells

**DOI:** 10.1039/c8ra04398a

**Published:** 2018-08-17

**Authors:** Francis Otieno, Mildred Airo, Rudolph M. Erasmus, David G. Billing, Alexander Quandt, Daniel Wamwangi

**Affiliations:** Material Physics Research Institute, School of Physics, University of the Witwatersrand Private Bag 3, Wits 2050 Johannesburg South Africa frankotienoo@gmail.com; School of Chemistry, University of the Witwatersrand Private Bag 3, Wits 2050 Republic of South Africa; Materials for Energy Research Group (MERG), University of the Witwatersrand Private Bag 3, 2050 Wits Johannesburg South Africa; Historical Museum of Physics and Study & Research Centre “Enrico Fermi” 00184 Roma Italy

## Abstract

Down conversion has been applied to minimize thermalization losses in photovoltaic devices. In this study, terbium-doped ZnO (ZnO:Tb^3+^) thin films were deposited on ITO-coated glass, quartz and silicon substrates using the RF magnetron sputtering technique fitted with a high-purity (99.99%) Tb^3+^-doped ZnO target (97% ZnO, 3% Tb) for use in organic solar cells as a bi-functional layer. A systematic study of the film crystallization dynamics was carried out through elevated temperature annealing in Ar ambient. The films were characterized using grazing incidence (XRD), Rutherford backscattering spectrometry (RBS), atomic force microscopy, and UV-visible transmittance and photoluminescence measurements at an excitation wavelength of 244 nm. The tunability of size and bandgap of ZnO:Tb^3+^ nanocrystals with annealing exhibited quantum confinement effects, which enabled the control of emission characteristics in ZnO:Tb^3+^. Energy transfer of ZnO → Tb^3+^ (^5^D_3_–^7^F_5_) was also observed from the photoluminescence (PL) spectra. At an inter-band resonance excitation of around 300–400 nm, a typical emission band from Tb^3+^ was obtained. The ZnO:Tb^3+^ materials grown on ITO-coated glass were then used as bi-functional layers in an organic solar cell based on P3HT:PCBM blend, serving as active layers in an inverted device structure. Energy transfer through down conversion between ZnO and Tb^3+^ led to enhanced absorption in P3HT:PCBM in the 300–400 nm range and subsequently augmented *J*_sc_ of a Tb^3+^-based device by 17%.

## Abstract 2

Thermal annealing of Tb doped ZnO thin films was undertaken and as proof of concept, pristine films were used as a bi-functional in inverted solar cell devices.

## Graphical abstract

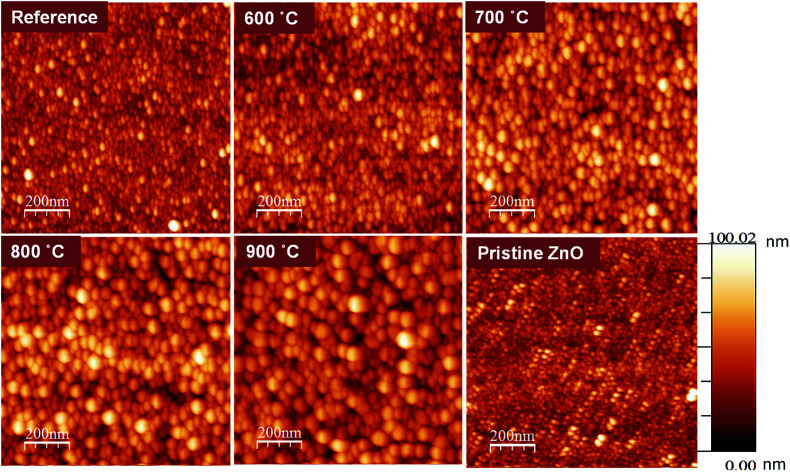

## 1. Introduction

Rare-earth (RE)-doped semiconductors have attracted significant interest for possible applications in high-power lasers, optical manipulation as visible emitting phosphors in display devices, and other optoelectronic products.^1,2^ This enormous interest for their application potential is due to stable intra-4f shell transitions of rare earth ions, which favor energy transfer from the host semiconductors to dopant RE ions.^3^ These transitions are shielded from their local environments by the completely filled 5s^2^ and 5p^6^ outer orbitals.

Zinc oxide (ZnO) thin films have continued to attract widespread research interest as transparent conducting oxides (TCOs) due to their high electrical conductivity (2 × 10^−6^ to 2 × 10^−4^ S cm^−1^)^4^ and optical transmission (>80% in the UV-visible range). This has been evidenced mainly in n-type ZnO due to the favorable formation energies of Zn^2+^/O^2−^ defects. Besides, ZnO has a wide and direct band gap (about 3.3 eV at room temperature) with large exciton binding energy (60 meV) and excellent chemical and thermal stabilities; it can be fabricated in a cost-effective and simple manner.^5^ These properties make it a good candidate for use as a transparent electrode in solar cells. Zinc oxide thin films can be grown using several methods such as magnetron sputtering, sol gel technique, spray pyrolysis, CVD, and PECVD.^6,7^ RF sputtering is usually preferred mainly due to its reproducibility, high deposition rate, low substrate temperature and ability to yield films with tunable preferred orientation.^8,9^

When doped with a rare earth metal, ZnO can be used as a sensitizer to excite rare-earth (RE) ions such as Ce^3+^, Er^3+^, Ho^3+^, Nd^3+^, Tm^3+^, Dy^3+^, Eu^3+^ and Tb^3+^. This is possible due to its large absorption cross-section and broad excitation spectrum.^10^ In this manner, RE can absorb the UV-blue emission from ZnO and emit in the visible or infra-red range, thus enabling this material to serve as an excellent doping host for rare earth ions with an optimal spectrum modifying matrix for diverse solar cells.

The optical properties of RE-doped ZnO usually depend on dopant concentrations and fabrication process as well as the host structure, which is sensitive to the crystal field energy and spin–orbit coupling. The emission characteristics of the rare earth ions in ZnO are sensitive to the nature of the fabrication process and the size of ZnO nano-crystals as well as their morphology. The morphology and structure of thin films can be tuned by changing the ad-atom energies and mobility through thermal treatment. Enhancing film crystallinity during growth due to lattice strains and thermal stresses between the ZnO film and the substrate is one approach for spectrum modification in these films.^1^

In organic solar cells, ZnO is widely used as a buffer layer since it exhibits low work function, excellent optical transparency, high electron mobility, and ease of fabrication.^11^ Combining the positive attributes of ZnO and RE in organic solar cells would therefore result in a good spectrum modifying matrix with dual functionality as an electron transporting layer and as a photon-conversion layer. In addition, the implementation of the buffer layer requires a simple architecture with no losses induced by the space gap between the down converting layer and active layers, which is a feature that is consistently persistent in externally stacked phosphor-based photovoltaic devices.

In this paper, the effects of annealing on the structure, morphology and optical properties of Tb^3+^-doped ZnO thin films fabricated at room temperature for photovoltaic applications through spectrum modification are reported. All films have been deposited using RF magnetron sputtering. As a proof of concept, ZnO and ZnO:Tb^3+^ thin films deposited on ITO glass have been incorporated as separate bi-functional layers in inverted organic solar devices. In these devices, the P3HT:PC_61_BM blend is used as the active layer of the bulk heterojunction (BHJ) in the following device architectures: glass/ITO/ZnO/P3HT:PCBM/PEDOT:PSS/Al and glass/ITO/ZnO:Tb^3+^/P3HT:PCBM/PEDOT:PSS/Al. The donor and acceptors in the bulk heterojunction are mixed in a mass ratio of 1 : 1. We report an increase in efficiency of 17.2%. This enhanced device performance may be due to the ZnO → Tb^3+^ energy transfer through the down-conversion process. All devices are fabricated and characterized at room temperature with exposure to air.

## 2. Experimental procedure

### 2.1. Materials

ZnO (97%) : Tb (3%) (99.99% purity) and ZnO disk targets of diameter 76 mm and thickness 6 mm sourced from Semiconductor Wafer, Inc. (SWI) have been used for thin film deposition. The Si wafer (001) and quartz substrates were also purchased from Semiconductor Wafer, Inc. (SWI). ITO (surface resistivity: 30–60 Ω sq^−1^), PEDOT:PSS (1.3 wt% dispersion in H_2_O), P3HT (purity: 99.995%) and PCBM (purity: 99.5%) were purchased from Sigma Aldrich.

### 2.2. The growth of ZnO:Tb^3+^ thin films

Tb^3+^-doped ZnO thin films were deposited at room temperature using RF magnetron sputtering onto n-type (001) silicon, ITO and quartz substrates. The substrates were initially cleaned with acetone and ethanol in an ultrasonic bath for 20 minutes and rinsed in deionized water before drying using a stream of dried nitrogen gas. They were then mounted on a rotating substrate holder at a distance of 6 cm from the target. The sputtering chamber was evacuated to a base pressure of about 2.5 × 10^−5^ mbar, and sputtering was performed under argon with a working gas pressure of 3.1 × 10^−3^ mbar. The growth was undertaken under 13 sccm Ar flow and 2.20 W cm^−2^ RF power density. Post-growth annealing was carried out in an Ar-filled furnace at a heating rate of 10 °C min^−1^ over a temperature range of 600–900 °C for 2 hours.

### 2.3. Characterization

The film morphologies and structures were characterized using Veeco Di-3100 atomic force microscopy (AFM) in the tapping mode. Grazing incidence X-ray diffraction (XRD) measurement of the ZnO:Tb^3+^ thin film was obtained using AXS Bruker D8 Discover, 40 kV, 40 mA using Cu Kα radiation in the Seeman–Bohlin geometry at a low scanning rate of 1.2° per minute at a glancing angle of 1°. The transmittance spectra of the films deposited onto the quartz substrates were measured using a Cary 500 UV-visible spectrophotometer. Photoluminescence measurements were obtained using a Horiba LabRAM HR spectrometer with a 150 lines per mm grating at an excitation wavelength of 244 nm from a frequency doubled Lexel argon ion laser. Rutherford backscattering spectrometry (RBS) measurements were performed to determine the composition and distribution profiles of Tb^3+^ ions in the ZnO matrix at room temperature using 4He^+^ particles of energy 1.6 MeV at a backscattering angle of 165° (IBM geometry). The beam current was maintained between 10 and 15 nA during measurements.

### 2.4. Device fabrication and characterization

The photovoltaic devices were fabricated on glass coated with indium tin oxide (ITO), which had been cleaned by sonication using the procedure described in ref. 12 and 13. Thin ZnO or ZnO:Tb^3+^ (about 35 nm) films were grown on the cleaned ITO by RF sputtering using the procedure described in Section 2.2 without annealing. Active layers composed of P3HT:PCBM blended at a ratio of 1 : 1 in chlorobenzene with a total concentration of 20 mg ml^−1^ were then spin-coated at 2000 rpm for 1 minute to form a film of thickness of 215 nm. The active layers were subsequently annealed at 80 °C for 10 min in an Ar-filled furnace before spin coating (80 nm) the hole transport layer (PEDOT:PSS). Finally, the Al electrode was thermally evaporated at a pressure of 2 × 10^−5^ Pa. The current density–voltage (*J–V*) characteristics were obtained using a HP 4141B source measure unit under 100 mW cm^−2^ illumination (AM 1.5G). All the measurements were carried out at room temperature under standard conditions. More than 20 devices of pristine ZnO- and ZnO:Tb^3+^-based inverted solar cells each of area 8.4 ± 0.1 mm^2^ were fabricated and characterized, and the results exhibited a high degree of repeatability to within 2.5%.

## 3. Results and discussions

### 3.1 Morphology studies

**Fig. 1:**
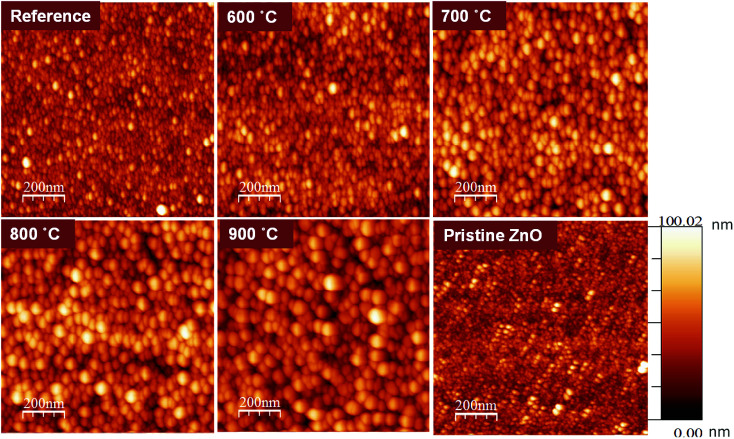
AFM images of pristine ZnO and ZnO:Tb^3+^ films (room temperature) and after annealing in an Ar-filled furnace for 2 hours in the range between 600 and 900 °C.

Fig. 1 shows the surface topographies of ZnO:Tb^3+^ and pristine ZnO films characterized using Veeco Di3100 AFM in the tapping mode.

The average roughness of the ZnO:Tb^3+^ film surface is shown in Table 1 for all temperature ranges of interest. As the annealing temperature increases, the surface roughness of the film increases dramatically (from 4.9 nm for the as-deposited ZnO:Tb^3+^ film to 36.9 nm at 900 °C).

**Table 1:** Summary of RMS and grain height from tapping mode AFM

Annealing temperature (°C)	Roughness RMS (nm)	Grain height (nm)
RT	4.9	12.2
600	11.8	49.3
700	17.2	54.0
800	27.8	68.8
900	36.9	81.4
ZnO	3.8	10.3

It is noted that the surface corrugations of ZnO:Tb^3+^ films analyzed from AFM could possibly represent the particle size and could thus be larger than the grain size determined from XRD, as shown in Table 2. This is plausible since the particle size measured from AFM is the surface morphology of coalesced grains.^14^ Furthermore, the geometry obtained from the XRD measurement of the grain size is normal to the surface of the film and not in plane. Diffusion during annealing also enables the atoms to occupy lattice sites, thereby enhancing film quality, as seen in the XRD data. The increase in grain height with annealing temperature is an indication of thermally activated diffusion of atoms that coalesce to larger grains in regions with lower activation barriers for self-diffusion.^15,16^ The major grain growth also results in an increase in the surface roughness, as evident by the RMS values. We also note a change in the surface morphology of the films due to terbium doping since the roughness of the surface increases compared to that of pristine ZnO at room temperature. This may be due to the accumulation of Tb^3+^ at the interstitial sites of the ZnO matrix. The emission of Tb^3+^, as seen in the PL spectra, cannot rule out this possibility.

**Table 2:** Characteristics of Tb^3+^-doped ZnO estimated from XRD measurements

Temperature (°C)	FWHM (100)	2 theta	Size, *D* (nm)	*a*/(Å)	*c*/(Å)	*δ* (10^16^)	*ε* (10^−3^)
Reference	1.620	31.397	5.09	3.28	5.69	3.86	6.81
600	1.473	31.901	5.61	3.23	5.61	3.18	6.18
700	1.332	31.784	6.19	3.24	5.63	2.63	5.59
800	1.332	31.764	6.20	3.25	5.63	2.60	5.57
900	1.207	31.820	6.84	3.25	5.64	2.14	5.07

### 3.2 RBS measurements

**Fig. 2:**
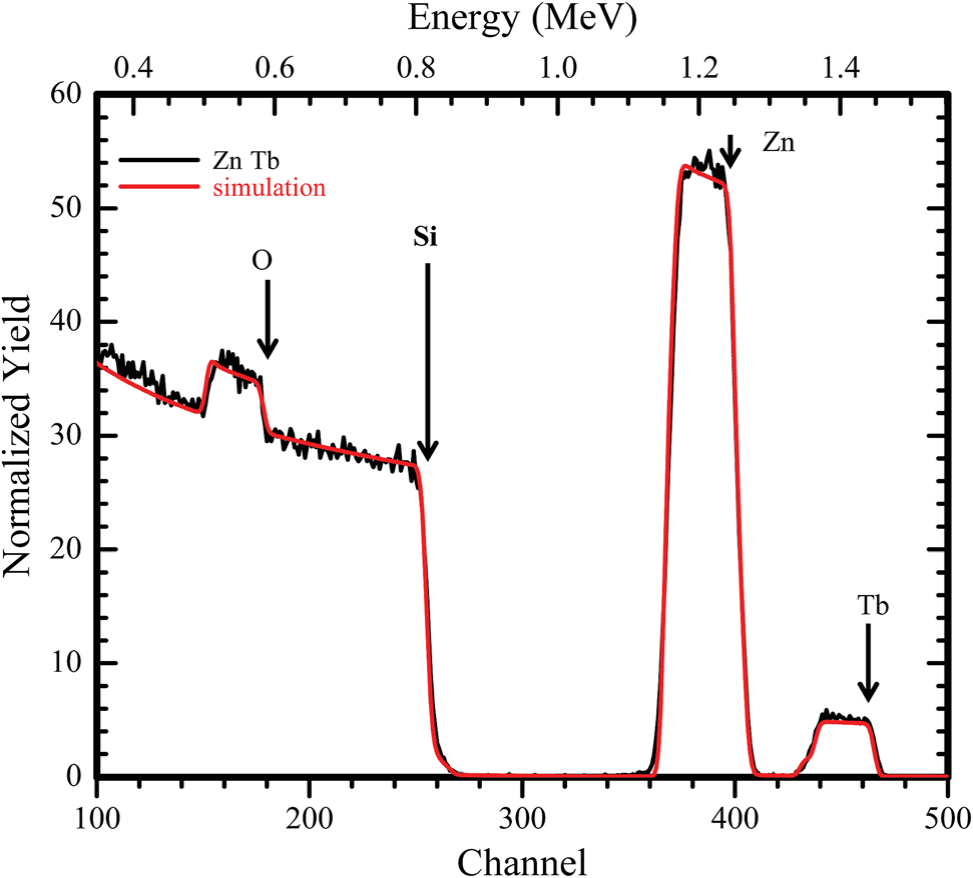
RBS spectrum of an 190 nm ZnO:Tb^3+^ layer on 200 nm Si showing experimental data and computer simulation using the XRump code. The sample structure and thicknesses of the layers were derived.

An RBS spectrum of ZnO:Tb^3+^ thin film on Si substrate annealed at 700 °C is shown in Fig. 2 together with a computer simulation using the XRump code. The experimental spectrum is well reproduced. The structure in the Si spectrum around channels 180, 260, 410 and 470 is due to the non-Rutherford scattering cross-section from O, Si, Zn and Tb, respectively. The surface energy positions in the channels of the elements have been shown using arrows. All other samples annealed at different temperatures show insignificant changes in the surface energy position and concentration.

The presence of Tb^3+^ in the films is very prominent, and the global atomic concentrations of different species contained in the film can be deduced. From the XRump simulation, the stoichiometry of the matrix with 48.1, 49.2 and 2.7% for Zn O and Tb, respectively, was derived. Tb^3+^ in channel 470 exhibited a nearly flat yield, suggesting homogeneous distribution of Tb^3+^ along the growth direction, which is independent of the annealing temperature.

### 3.3 Grazing incidence XRD measurements

**Fig. 3:**
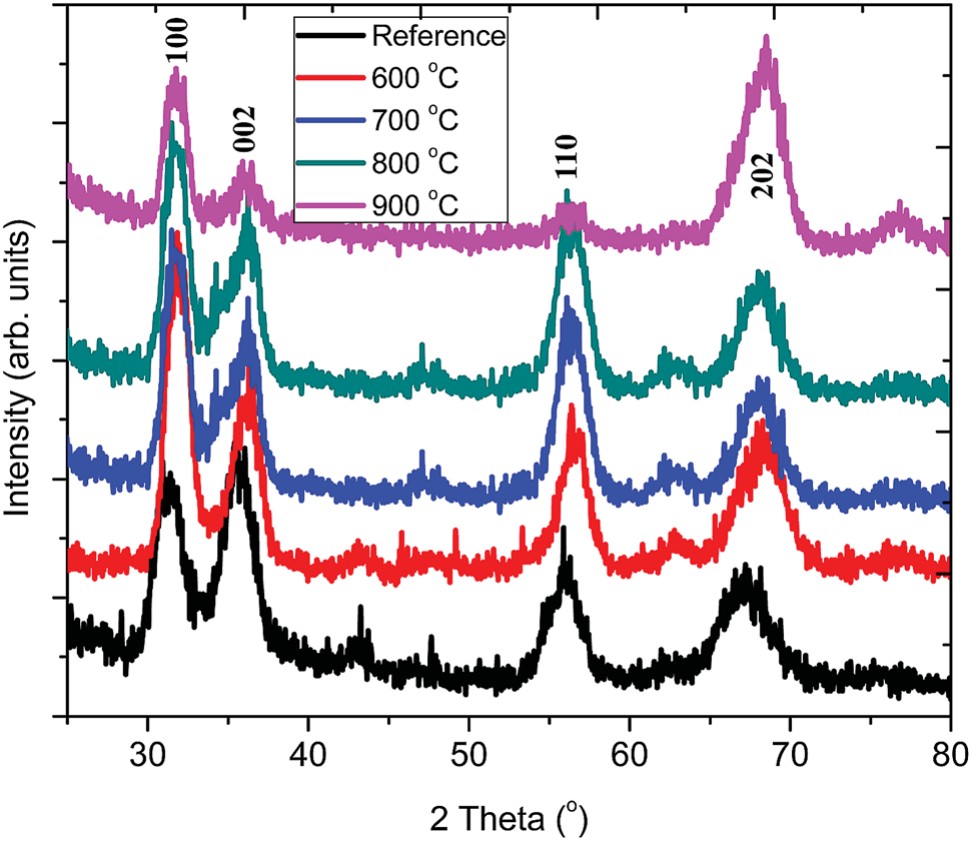
Grazing incidence X-ray diffraction of ZnO:Tb^3+^ thin films deposited by RF sputtering at room temperature followed by annealing at different temperatures (600–900 °C).

Fig. 3 shows the grazing incidence XRD patterns of pristine ZnO:Tb^3+^ and those obtained after annealing in the temperature range of 600–900 °C in an Ar-filled furnace. It can be observed that the pristine ZnO:Tb^3+^ film is partially crystalline with low intensity of the *hkl* reflex. Annealing leads to preferred orientation of the ZnO reflexes. The typical strong diffraction peaks ascribed to hexagonal wurtzite ZnO (JCP2) (card number, 003-0888) are observed without any additional peaks from other oxide phases at elevated temperatures. This is an indication that the films are of high phase purity and that no diffraction peaks corresponding to Tb^3+^-based compounds or any other impurities are present. The diffraction peaks become gradually stronger up to 800 °C, which is an indication of increasing crystallinity. The appearance of reduced peak intensity and dislocation density after annealing at 900 °C may be due to the presence of porosity.^17^ The dislocation density (*d*), defined as the length of dislocation lines per unit volume of the crystal (Williamson and Smallman), and the strain present in the thin film are also investigated and presented in Table 2.

FWHM generally decreases as the annealing temperature increases, thus leading to the growth of Tb^3+^-doped ZnO nanocrystals from 5.09 nm without annealing to 6.84 nm upon annealing at 900 °C. This is because annealing increases adatom mobility and thus reduces compressive stress and structural defects occurring due to disorder in the pristine ZnO:Tb^3+^ films; this leads to enhanced crystallinity. As the annealing temperature increases, the compressive residual stress is converted gradually to the tensile residual stress in the films due to the interaction between thermal stress and lattice stress.^18^ The small crystallites coalesce to larger crystallites, thus increasing the grain size with the increasing annealing temperature. The competition between the long-range Coulomb attractive and short-range repulsive interactions in ionic nanocrystals creates an effective negative pressure, which can decrease the lattice parameters *a* and *c* upon annealing.^19^ At low temperature, the difference in the thermal expansion coefficients of ZnO (6.05 and 3.53 × 10^−6^/°C for *α*_11_ and *α*_33_, respectively, for the hexagonal structure) and Si (2.5 × 10^−6^/°C at room temperature) causes an increase in the tensile stress generated by silicon when the substrate temperature drops from a high temperature to room temperature; this cancels out the compressive stress of ZnO. However at high temperatures (600–900 °C) used here, the tensile stress generated by the substrate overcomes the compressive stress of ZnO, causing a shift of the peaks to high angles, as seen in Fig. 3.^20^ However, the lattice constants *a* and *c* increase gradually, thus suggesting that larger Tb^3+^ (ionic radius 0.118 nm)^21^ substitutes smaller Zn^2+^ (the radius of Zn^2+^ ion is 0.074 nm).^17^ This increase is marginal within the 600–900 °C temperature range and indicates that the thermal stress within the film is independent of the substrate.

### 3.4 Optical properties

**Fig. 4:**
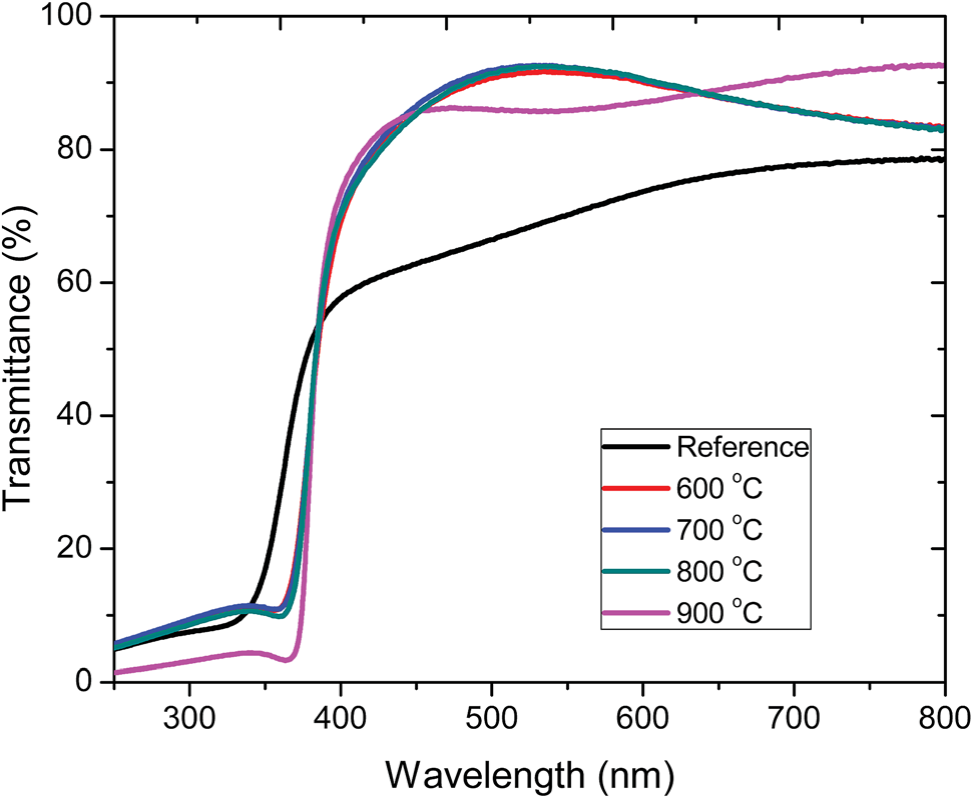
Transmission spectra of pristine ZnO:Tb^3+^ thin films and upon annealing at different temperatures 600–900 °C in an Ar-filled furnace.

Fig. 4 shows the optical transmittance of pristine (183 nm) and annealed (190 nm) ZnO:Tb^3+^ films on quartz substrates in the wavelength range of 250–800 nm. The sharp optical transmission edge observed around 380 nm corresponds to the inter-band transitions of the valence electrons, which is consistent with the laser excitation energy used in the PL measurements. The contributions of the substrate have been de-convoluted from the transmission spectra through calibration using the substrates as the reference.

The average transmittance for all the deposited films is between 71% and 92% in the visible part of the spectrum, thus enabling the use of these ZnO:Tb^3+^ films in thin film solar cell applications.^22^ At short wavelengths, a sharp decrease in the transmittance is observed around the absorption edge. The enhanced absorption at 900 °C is ascribed to increased crystallinity, leading to a decrease in the extinction coefficient as structural defects are minimized with the increasing temperature, thus reducing the absorption coefficient.^23^ To correlate the effect of temperature and grain size with the optical properties of ZnO:Tb^3+^ thin films, the absorption coefficient (*α*) and the band gap (*E*_g_) were determined from Tauc's formalism for direct and indirect transitions in the form(*αhν*) = *A*(*hν* − *E*_g_)^*p*^where *A* is a constant, *h* is the Plank's constant and *p* is an exponent that is dependent on the nature of the allowed or forbidden electronic transitions. Thus, for allowed direct transition, *p* = 1/2, whereas *p* = 3/2 describes the case for forbidden direct transition; *p* = 2 indicates indirect allowed transition, and *p* = 3 indicates indirect forbidden transitions. Since ZnO:Tb^3+^ is a direct band gap semiconductor with a hexagonal wurtzite crystal structure, as confirmed in the XRD measurements, *p* = 1/2.^24–26^*hν* is the photon energy expressed in eV, whereas *α* is the absorption coefficient, and *α* is calculated using the relation*T* = exp(−*αd*)where *T* is the optical transmittance and *d* is the thickness of ZnO:Tb^3+^ films.

**Fig. 5:**
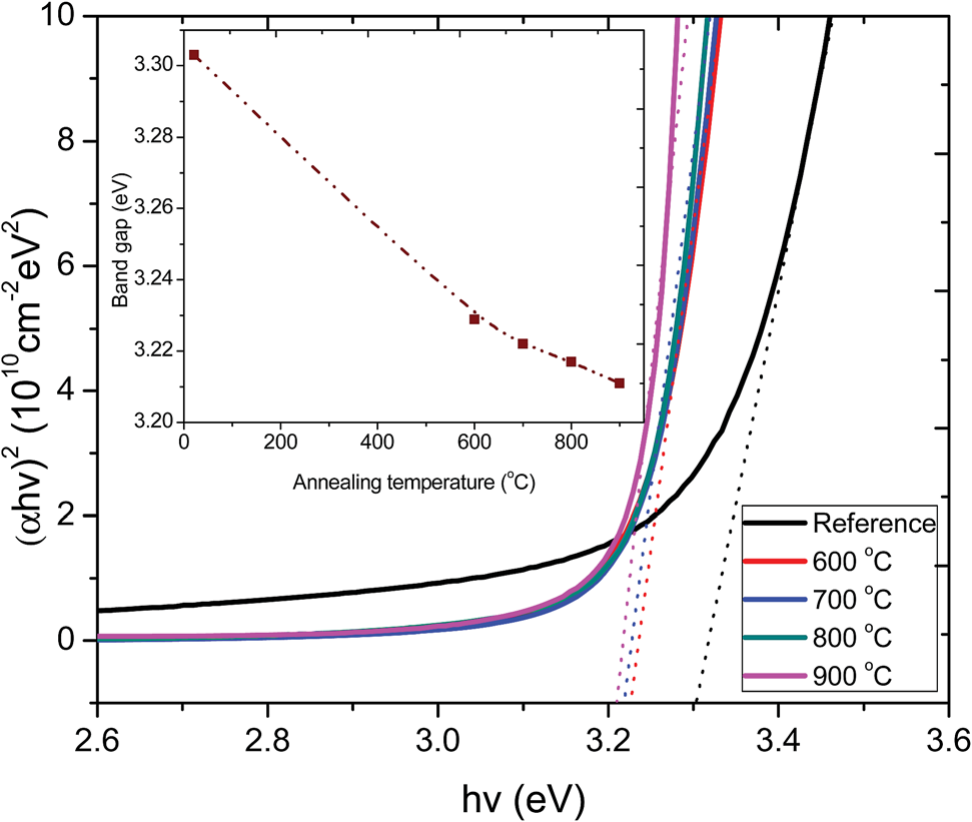
Plot of (*αhν*)^2^*versus hν* for the pristine ZnO:Tb^3+^ thin film and after annealing at 600–900 °C. The inset shows the variation of optical band gap as a function of annealing temperature.

The optical band gaps of the films annealed at various temperatures were obtained by plotting (*αhν*)^2^ as a function of photon energy (*hν*). The values of the direct energy gap, *E*_g_, were derived from the intercept of the extrapolation to zero absorption with the photon energy axis, as shown in Fig. 5. The non-zero absorbance below the energy cut-off for the pristine film was ascribed to phonon contributions associated with structural defects or trap states arising from a partially crystalline pristine ZnO:Tb^3+^ film.

The sharpness of the absorption edge is found to be maximum at an annealing temperature of 800 °C; beyond this temperature, it broadens. The change in the optical band gap is comparatively small, but a minimum is seen at 900 °C. The estimated band gap values for all the samples are summarized in the inset figure in Fig. 5. These values are within the range reported for films and single crystal^27–29^ and are also in agreement with band gap approximation obtained from the PL results.

### 3.5 Photoluminescence studies

**Fig. 6:**
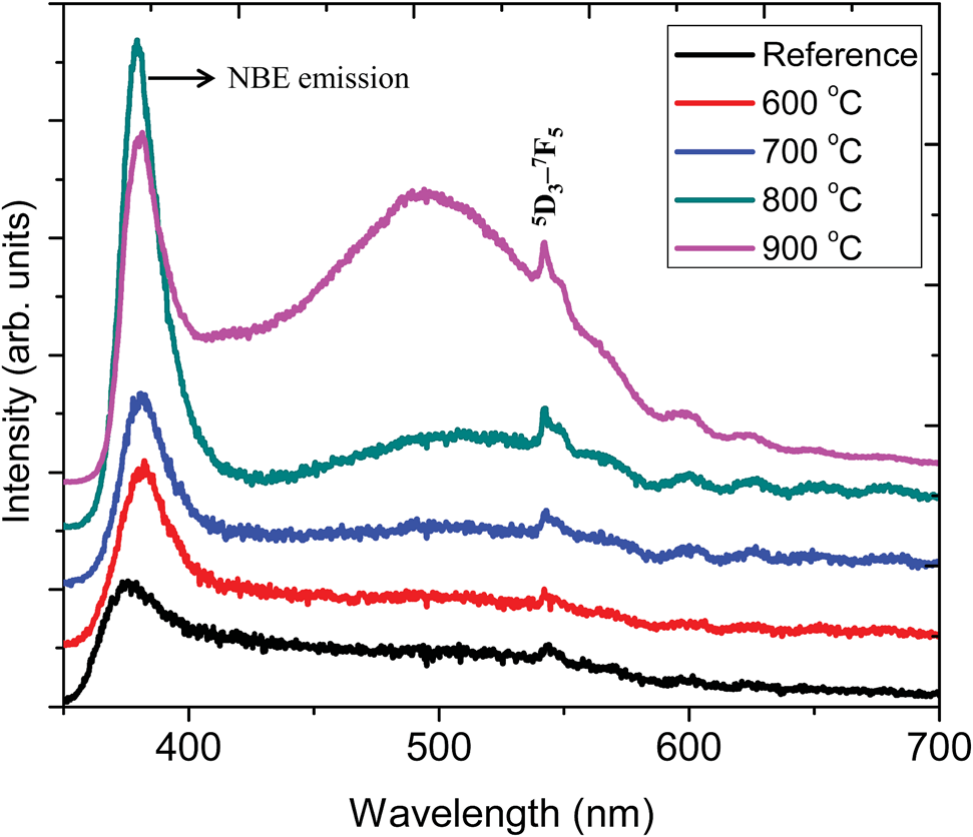
PL spectra of the ZnO:Tb^3+^ thin films annealed at different temperatures and excited at 244 nm together with that of the ZnO:Tb^3+^ thin film annealed at temperature range of 600–900 °C.

Fig. 6 shows the PL emission spectrum of ZnO:Tb^3+^ thin film excited at 244 nm. The spectrum shows an emission at 378 nm corresponding to a band-to-band emission of ZnO. This emission is due to the recombination of the excitons of granular ZnO through an exciton–exciton collision process, corresponding to near band edge (NBE) emission of ZnO;^30^ its intensity increases with the increasing annealing temperature, which is an indication of the enhanced film crystallinity necessary for effective charge transfer (grain size commensurate to the mean free path of the electrons) in solar cell applications. This is due to the decrease in non-radiative defects and the increase in ZnO grain size.^1^ At 900 °C, this peak shows reduced peak intensity. A plausible reason is that the decrease in the length of the *c*-axis of ZnO films annealed at higher temperature may induce an acute lattice distortion, which can significantly increase the polarizability in the ZnO:Tb^3+^ matrix, thus affecting the optical properties of ZnO:Tb^3+^ films and quenching the PL efficiency.^31^ This is in agreement with XRD findings. This decrease in the emission of ZnO or the inter-band transitions can also be ascribed to some of the excited electrons being transferred to the energy levels associated with oxygen vacancies, whereas others are transferred to the energy levels of Tb^3^. The broad emissions between 400 and 500 nm are due to deep level defect emissions, arising from the recombination between Zn_*i*_ and the valence band level.^32^ ZnO has six kinds of defects classified as vacancies, interstitials and antisites, and their emission intensities can be influenced by the defect concentration and defect–defect interactions, which vary with the annealing temperature.

The sharp peak at 543 nm is related to emission from Tb^3+^ ions. Here, the electric dipole (ED) transitions between the 4f states in the free Tb^3+^ ions are parity forbidden. However the ED transitions are partially allowed, but they have weak intensity when Tb^3+^ ions occupy interstitial or lattice sites in ZnO, which is a condensed matter, and a large absorption transition probability arises from its direct band gap nature.^32,33^ Hence, according to Bylander *et al.*,^34^ the majority of excited charge carriers trapped at Tb^3+^ centers originate from band gap absorption in the ZnO matrix, and a marginal fraction of these electrons are due to the 4f–4f absorption transition within the Tb^3+^ ions.

Table 3 shows the peak centroid and the corresponding full width at half maximum (FWHM) for the near band edge peak as well as for the broad band peak, whereas Fig. 8 shows an elaborate schematic band diagram for these proposed emissions. The shift of the peak center with annealing temperature, shown in Table 3, can be due to the relaxation of the built-in strain in ZnO:Tb^3+^ thin films.

**Table 3:** Summary of peak center position and FWHM for the NBE PL peak

Annealing temperature (°C)	Peak centre (nm)	FWHM (nm)
Reference	477	21.9
600	381	19.1
700	382	16.6
800	384	12.2
900	398	26.3

**Fig. 7:**
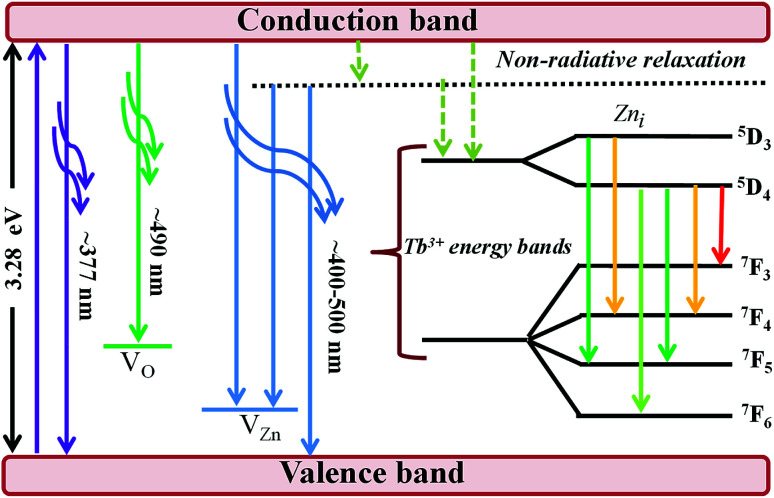
Schematic of the energy level diagram of the ZnO:Tb^3+^ thin films.

The efficiency of the energy transfer from the host ZnO to rare earth ions such as Tb^3+^ is usually low due to the large mismatch in the ionic radii and the valence states of Zn^2+^ (74 pm) and rare earth ions such as Tb^3+^ (118 pm). Furthermore, the location of the rare earth ions in the host also determines the efficiency of the energy transfer; thus, generally, rare earth ions can occupy two different sites in the hosts, namely, (i) the substitution sites of Zn^2+^ in the ZnO lattice or (ii) the ZnO grain boundaries. The location of Tb^3+^ on the surface or grain boundaries can be ruled out from the RBS data, which consistently show a relatively homogeneous distribution of Tb^3+^ in the ZnO thin film. The distinction of surface and bulk emissions can thus be resolved qualitatively using the RBT profiles of the RE and the film constituent atoms. Fig. 7 shows a schematic diagram of the energy levels in the ZnO:Tb^3+^ thin films.

All samples show defect-related emissions with a major green emission peak at 543 nm and a few oscillations due to the emissions of Tb^3+^, which occur at 492, 543, 595, 624, 543 and 563 nm; these peaks represent the ^5^D_4_–^7^F_6_, ^5^D_4_–^7^F_5_, ^5^D_4_–^7^F_4_, ^5^D_4_–^7^F_3_, ^5^D_3_–^7^F_5_ and ^5^D_3_–^7^F_4_ transitions of Tb^3+^, respectively. The excitations are assigned to direct excitation of Tb^3+^ through f → d transitions. These transitions are assigned according to.^35,36^ A band gap value of 3.28 eV is obtained from the NBE peak. The theoretical position of the Zn_*i*_ level is at 0.22 eV below the conduction band.^34^ With excitation at 244 nm, charge carriers are pumped from the valence band to the conduction band leaving a hole in the VB and an electron in the CB, which can radiatively recombine to give UV emission; this is generally assigned to the near band edge emission (377 nm) from ZnO, which is shown in Fig. 7.

### 3.6 *I*–*V* characteristics

**Fig. 8:**
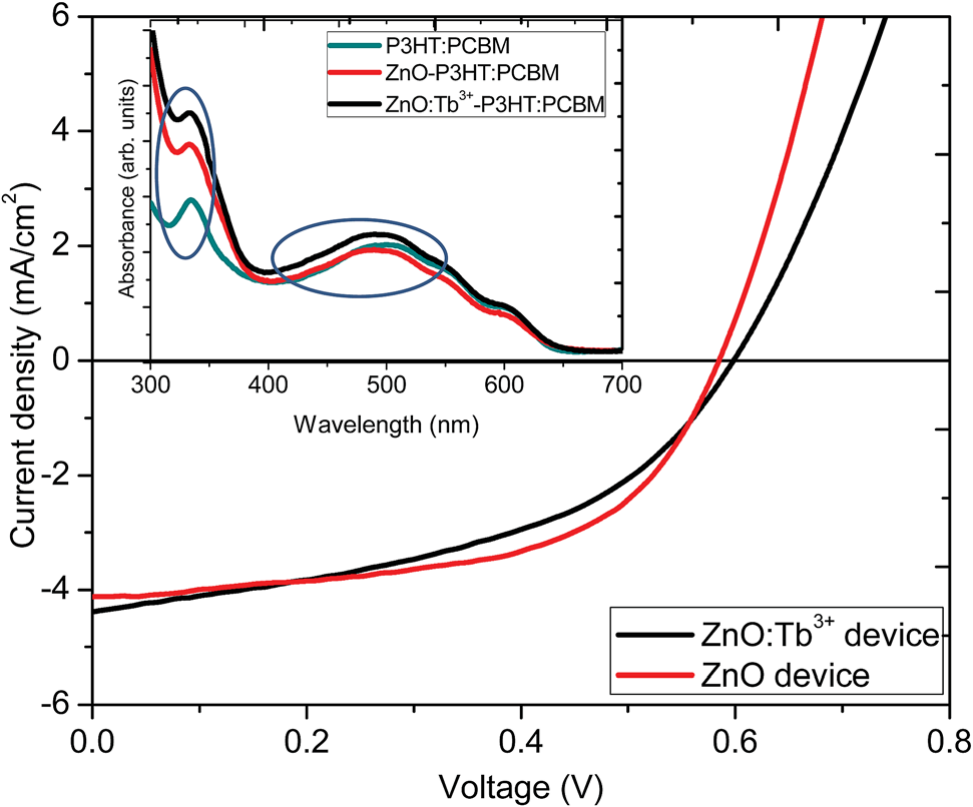
*J*–*V* characteristics of devices formed using ZnO and ZnO:Tb^3+^ as the cathode layers. Inset is the UV-visible absorption spectra of the active layer with different cathode layers.

Fig. 8 shows *J*–*V* characteristic curves as a proof of concept for the influence of Tb^3+^ doping on the photoactive performance in organic solar cells with an inverted structure of ITO/ZnO:Tb^3+/^P3HT:PCBM/PEDOT:PSS/Al. The inset in Fig. 8 represents the UV-visible absorption spectra of active layer (P3HT:PCBM), ZnO/P3HT:PCBM and ZnO:Tb^3+^/P3HT:PCBM films used in device fabrication. The films were used without annealing to avoid softening of the glass as well as deterioration of the electrical properties of the ITO film.

ZnO and ZnO:Tb^3+^ thin films grown by RF sputtering on ITO-coated glass substrates were used as both electron transport and spectral conversion layers. Table 4 shows the performance parameters. The two devices showed almost similar fill factor (FF) and open circuit voltage (*V*_oc_) values, which indicated that the electronic properties of ZnO nanocrystals were not altered by Tb^3+^ doping. This was also supported by the nearly constant value of the shunt resistance of the device. However, there was a slight increase in *J*_sc_ from 4.11 to 4.82 mA cm^−2^. This could be due to spectral down-conversion as a result of Tb^3+^ doping. According to the absorption measurements, shown in the inset in Fig. 8, there was an increase in absorption in the ultraviolet region from 300 to 400 nm with the introduction of a layer of ZnO thin film before the P3HT:PCBM blend thin film, which further enhanced upon doping with Tb^3+^, leading to an increase in the number of photo-generated photons in the visible region converted from the UV region. The down-conversion process altered the UV properties of the solar cell under the solar emission spectrum, enabling the absorption of photons in the UV range and re-emission at longer wavelengths in the visible region, where the organic solar cell exhibited a considerably better response. The increase in light absorption with the ZnO thin film compared to that for the film with the P3HT:PCBM blend alone may be an indication that the emission is in resonance with the P3HT electronic structure since exciton generation occurred through π → π* interaction in P3HT.

**Table 4:** Summary of the performance of the P3HT:PCBM bulk heterojunction solar cell devices with pure ZnO and ZnO:Tb^3+^ as the cathode buffer layers

Device	*J*_sc_ (mA cm^−2^)	*V*_oc_ (V)	*R*_s_ (Ω cm^2^)	*R*_sh_ (kΩ cm^2^)	FF (%)	PCE (%)
ZnO device	4.11 ± 0.10	0.59 ± 0.02	51.8 ± 1.2	232 ± 3	47.8 ± 1.0	1.16 ± 0.08
ZnO:Tb^3+^ device	4.82 ± 0.12	0.60 ± 0.01	52.3 ± 1.0	235 ± 2	46.9 ± 1.1	1.36 ± 0.06

## 4. Conclusion

Polycrystalline ZnO:Tb^3+^ thin films with high orientation were deposited on Si, quartz and ITO-coated glass substrates by the RF magnetron sputtering technique. The films showed excellent transparency and conductivity, which are required for solar cell applications. X-ray diffraction (XRD), atomic force microscopy (AFM) and the photoluminescence (PL) were employed to analyze the influence of post-deposition annealing treatments on the structural properties of the ZnO:Tb^3+^ thin films. The films showed an increasing roughness and grain size of ZnO:Tb^3+^ with annealing temperatures between 600 and 900 °C and a shift in the diffraction peak position. As a proof of concept, hybrid OPV devices based on the P3HT:PCBM blend were fabricated. The incorporation of ZnO and ZnO:Tb^3+^ thin films resulted in an increase in absorption within the 300–400 nm wavelength range. The high absorption obtained when ZnO:Tb^3+^ was used showed that the films can be effectively used as a buffer layer and down-conversion layer, which resulted in enhancement in the OPV device performance.

## Conflicts of interest

There are no conflicts to declare.

## Supplementary Material

## References

[cit1] Teng X., Fan H., Pan S., Ye C., Li G. (2006). Influence of annealing on the structural and optical properties of ZnO: Tb thin films. J. Appl. Phys..

[cit2] Matsumoto T., Kato H., Miyamoto K., Sano M., Zhukov E. A., Yao T. (2002). Correlation between grain size and optical properties in zinc oxide thin films. Appl. Phys. Lett..

[cit3] Lang J., Wang J., Zhang Q., Han Q., Yang J., Xu S., Wang D., Wei M., Li X., Sui Y. (2016). Tunable bandgap and optical properties of (Eu, Sm) codoped ZnO nanoparticles. J. Mater. Sci.: Mater. Electron..

[cit4] Caglar M., Ilican S., Caglar Y., Yakuphanoglu F. (2009). Electrical conductivity and optical properties of ZnO nanostructured thin film. Appl. Surf. Sci..

[cit5] Kumar G. A., Reddy M. R., Reddy K. N. (2013). Structural, optical and electrical characteristics of nanostructured ZnO thin films with various thicknesses deposited by RF magnetron sputtering. Res. J. Phys. Sci..

[cit6] Xie H.-p., Zhang S.-r., Yang C.-t., Zhang H.-w., Yang Y. (2008). Influence of Postdeposition Annealing on the Properties of ZnO Films Prepared by RF Magnetron Sputtering. Piezoelectr. Acoustoopt..

[cit7] Mosbah A., Moustaghfir A., Abed S., Bouhssira N., Aida M., Tomasella E., Jacquet M. (2005). Comparison of the structural and optical properties of zinc oxide thin films deposited by dc and rf sputtering and spray pyrolysis. Surf. Coat. Technol..

[cit8] Water W., Chu S.-Y. (2002). Physical and structural properties of ZnO sputtered films. Mater. Lett..

[cit9] Shimomura T., Kim D., Nakayama M. (2005). Optical properties of high-quality ZnO thin films grown by a sputtering method. J. Lumin..

[cit10] Muth J., Kolbas R., Sharma A., Oktyabrsky S., Narayan J. (1999). Excitonic structure and absorption coefficient measurements of ZnO single crystal epitaxial films deposited by pulsed laser deposition. J. Appl. Phys..

[cit11] Wu N., Luo Q., Qiao X., Ma C.-Q. (2015). The preparation of a Eu3+-doped ZnO bi-functional layer and its application in organic photovoltaics. Mater. Res. Express.

[cit12] Otieno F., Mutuma B. K., Airo M., Ranganathan K., Erasmus R., Coville N., Wamwangi D. (2017). Enhancement of organic photovoltaic device performance via P3HT: PCBM solution heat treatment. Thin Solid Films.

[cit13] Otieno F., Airo M., Ranganathan K., Wamwangi D. (2016). Annealed silver-islands for enhanced optical absorption in organic solar cell. Thin Solid Films.

[cit14] Fang Z., Yan Z., Tan Y., Liu X., Wang Y. (2005). Influence of post-annealing treatment on the structure properties of ZnO films. Appl. Surf. Sci..

[cit15] Shih W.-C., Wu M.-S. (1994). Growth of ZnO films on GaAs substrates with a SiO2 buffer layer by RF planar magnetron sputtering for surface acoustic wave applications. J. Cryst. Growth.

[cit16] Puchert M., Timbrell P., Lamb R. (1996). Postdeposition annealing of radio frequency magnetron sputtered ZnO films. J. Vac. Sci. Technol., A.

[cit17] Otieno F., Airo M., Erasmus R. M., Billing D. G., Quandt A., Wamwangi D. (2017). Structural and spectroscopic analysis of ex situ annealed RF sputtered aluminium doped zinc oxide thin films. J. Appl. Phys..

[cit18] Wang M., Wang J., Chen W., Cui Y., Wang L. (2006). Effect of preheating and annealing temperatures on quality characteristics of ZnO thin film prepared by sol–gel method. Mater. Chem. Phys..

[cit19] Perebeinos V., Chan S.-W., Zhang F. (2002). ‘Madelung model’ prediction for dependence of lattice parameter on nanocrystal size. Solid State Commun..

[cit20] Chu S.-Y., Water W., Liaw J.-T. (2003). Influence of postdeposition annealing on the properties of ZnO films prepared by RF magnetron sputtering. J. Eur. Ceram. Soc..

[cit21] Li L., Li G., Che Y., Su W. (2000). Valence Characteristics and Structural Stabilities of the Electrolyte Solid Solutions Ce1-x RE x O2-δ (RE= Eu, Tb) by High Temperature and High Pressure. Chem. Mater..

[cit22] Oh B.-Y., Jeong M.-C., Lee W., Myoung J.-M. (2005). Properties of transparent conductive ZnO: Al films prepared by co-sputtering. J. Cryst. Growth.

[cit23] Gupta V., Mansingh A. (1996). Influence of postdeposition annealing on the structural and optical properties of sputtered zinc oxide film. J. Appl. Phys..

[cit24] Wahab H., Salama A., El-Saeid A., Nur O., Willander M., Battisha I. (2013). Optical, structural and morphological studies of (ZnO) nano-rod thin films for biosensor applications using sol gel technique. Results Phys..

[cit25] Chen J., Chen J., Chen D., Zhou Y., Li W., Ren Y., Hu L. (2014). Electrochemical deposition of Al-doped ZnO transparent conducting nanowire arrays for thin-film solar cell electrodes. Mater. Lett..

[cit26] Kang B., Ren F., Heo Y., Tien L., Norton D., Pearton S. (2005). pH measurements with single ZnO nanorods integrated with a microchannel. Appl. Phys. Lett..

[cit27] Yoshikawa H., Adachi S. (1997). Optical constants of ZnO. Jpn. J. Appl. Phys..

[cit28] Tansley T., Neely D., Foley C. (1983). Optical dispersion in zinc oxide. Phys. Status Solidi A.

[cit29] Heiland G., Mollwo E., Stöckmann F. (1959). Electronic processes in zinc oxide. Solid State Phys..

[cit30] Kong Y., Yu D., Zhang B., Fang W., Feng S. (2001). Ultraviolet-emitting ZnO nanowires synthesized by a physical vapor deposition approach. Appl. Phys. Lett..

[cit31] Lee Y.-C., Hu S.-Y., Water W., Tiong K.-K., Feng Z.-C., Chen Y.-T., Huang J.-C., Lee J.-W., Huang C.-C., Shen J.-L. (2009). Rapid thermal annealing effects on the structural and optical properties of ZnO films deposited on Si substrates. J. Lumin..

[cit32] Kumar V., Ntwaeaborwa O. M., Swart H. C. (2016). Deep level defect correlated emission and Si diffusion in ZnO: Tb 3+ thin films prepared by pulsed laser deposition. J. Colloid Interface Sci..

[cit33] Liu S.-M., Liu F.-Q., Wang Z.-G. (2001). Relaxation of carriers in terbium-doped ZnO nanoparticles. Chem. Phys. Lett..

[cit34] Bylander E. (1978). Surface effects on the low-energy cathodoluminescence of zinc oxide. J. Appl. Phys..

[cit35] Wang D., Yang P., Cheng Z., Wang W., Hou Z., Dai Y., Li C., Lin J. (2012). Patterning of Gd 2 (WO 4) 3: Ln 3+(Ln= Eu, Tb) luminescent films by microcontact printing route. J. Colloid Interface Sci..

[cit36] Kumar V., Ntwaeaborwa O., Coetsee E., Swart H. (2016). Role of deposition time on the properties of ZnO: Tb 3+ thin films prepared by pulsed laser deposition. J. Colloid Interface Sci..

